# Comparison of thoracic ultrasonography and thoracic radiography between healthy adult horses and horses with bacterial pneumonia using a novel, objective ultrasonographic scoring system

**DOI:** 10.3389/fvets.2022.991634

**Published:** 2022-10-13

**Authors:** Kate L. Hepworth-Warren, Nathan Nelson, Katarzyna A. Dembek, Kimberly A. S. Young

**Affiliations:** ^1^Department of Clinical Sciences, College of Veterinary Medicine, North Carolina State University, Raleigh, NC, United States; ^2^Department of Molecular Biomedical Sciences, College of Veterinary Medicine, North Carolina State University, Raleigh, NC, United States

**Keywords:** equine pneumonia, thoracic radiography, imaging, equine pleuropneumonia, ultrasonography

## Abstract

**Background:**

Thoracic ultrasonography (TUS) is widely used in equine practice but comparison to radiography is limited in horses.

**Objectives:**

To validate a novel, objective scoring system for TUS in adult horses and to compare ultrasonographic and radiographic findings.

**Animals:**

13 healthy horses and 9 with confirmed bacterial pneumonia

**Methods:**

Prospective study in which TUS and radiography were performed on healthy horses and those with bacterial pneumonia confirmed by clinical signs and results of transtracheal wash analysis. Ultrasonography was scored utilizing a novel scoring system evaluating number of comet tail lesions, the presence or absence of pleural effusion and/or pulmonary consolidation in each intercostal space. Eighteen horses had thoracic radiographs taken that were scored by a board-certified radiologist utilizing a previously described system. Total scores were recorded and compared between control and diseased patients.

**Results/Findings:**

Ultrasonographic scores were significantly higher in the diseased group (median= 126) than in the control group (median = 20, *p* = 0.01). Receiver operating characteristics (ROC) analysis identified a sensitivity of 66.7% (95% CI 0.417–1) and specificity of 92.3% (95% CI 0.462–1) for the ability of ultrasonography to identify bacterial pneumonia utilizing a TUS score cutoff of 37.

**Conclusions and clinical importance:**

TUS had moderate sensitivity and high specificity for identification of bacterial pneumonia in adult horses. TUS appears to be an acceptable stand-alone imaging modality for diagnosis of bacterial pneumonia in horses when radiography is not practical.

## Introduction

Thoracic ultrasonography (TUS) is widely used in equine medicine for the diagnosis of bacterial pneumonia due to the simplicity of use, its non-invasive nature, and the high availability of portable machines for field use. In an ambulatory practice setting, thoracic radiography of the adult horse is often not possible, thus TUS is relied upon heavily in the evaluation of horses with signs of bacterial pneumonia (1).

Thoracic ultrasonography in human and small animal medicine is comparable in sensitivity to radiography and computed tomography (CT) for the diagnosis of pneumonia, pulmonary edema, pneumothorax and pulmonary contusions (2–6). While there have been direct comparisons between the sensitivity of TUS and radiography in the identification of *Rhodococcus equi* pneumonia in foals and pneumothorax in the adult horse, the two modalities have not been compared for their diagnostic accuracy in bacterial pneumonia in adult horse (7, 8).

In order to compare ultrasonographic and radiographic images, previous studies in humans, small animals, dairy calves and foals have utilized objective scoring systems (3–6, 9–16). Numerous ultrasonographic and radiographic scores have been proposed for the evaluation of pleural changes associated with *Rhodococcus equi* pneumonia in foals, asthma and exercise-induced pulmonary hemorrhage in adult horses, bronchopneumonia in calves, pulmonary injury in dogs, and pneumonia and pulmonary edema in humans (3–6, 9–18). In addition to providing an objective measure of comparison between different modalities, these scoring systems have been applied clinically in disease screening and to expedite diagnostics and therapy including assessing severity and progression of pneumonia in humans with COVID-19 (11, 19, 20).

An objective scoring system for TUS in adult horses that accounts for the presence of pleural effusion and pulmonary consolidation, in addition to comet tails, has not previously been compared to thoracic radiography. The objectives of this study were to develop a novel scoring system for TUS in adult horses and to compare it to thoracic radiography utilizing a scoring system developed by Mazan et al. and to compare the TUS score and radiographic score in healthy horses to horses diagnosed with bacterial pneumonia (21). We hypothesized that this ultrasonographic scoring system would be an accurate and simple method of assessment of TUS in adult horses, and that ultrasonographic scores would correlate with radiographic scores and be a sensitive diagnostic for the identification of bacterial pneumonia. We hypothesized that horses classified as healthy based on history, physical examination and normal complete blood count would have significantly lower TUS and radiographic scores than those diagnosed with bacterial pneumonia.

## Materials and methods

### Patient selection and grouping

This was a prospective case-control study that included clinical patients presented to the (NC State College of Veterinary Medicine Veterinary Teaching Hospital) between August of 2020 and September of 2021. Client consent was obtained for the use of each animal, and all protocols were approved by the Institutional Animal Care and Use Committee. Control animals were horses of three or more years of age that were presented for elective magnetic resonance imaging for orthopedic disease and deemed healthy based on physical examination, rebreathing examination and complete blood count. Physical examination was considered normal if vital parameters were within normal limits (temperature <101.5°F, heart rate 28–48 beats per min, respiration 10–20 breaths per min) and there was no nasal discharge, cough, abnormal lung sounds on auscultation, or increased respiratory effort. Rebreathing exam was considered normal if there was no coughing noted during or after the exam and if the horse returned to a normal resting respiratory rate in fewer than 6 breaths after removal of the rebreathing bag. Exclusion criteria for control animals included history of respiratory or cardiac disease, and any of the following within the 90 days prior to the study: transport of >6 consecutive hours, esophageal obstruction, or general anesthesia. Horses in the diseased group were clinical patients of three or more years of age that were enrolled following diagnosis of bacterial pneumonia. Pneumonia was diagnosed by the attending clinician *via* clinical signs, clinicopathologic data and thoracic imaging, and was confirmed by transtracheal wash cytology (confirming intracellular bacteria and suppurative inflammation) and/or positive bacterial culture of transtracheal wash fluid. All animals in the control group and 6 horses in the diseased group had ultrasonographic exams and right to left lateral radiography performed of the lungs within 6 h of each other.

### Scoring of radiographic findings

Radiographs were assessed by a board-certified radiologist (NN who was blinded to the ultrasonographic findings) and the group assignment of each patient. Each series was scored according to the system previously described in detail by Mazan et al. (21). The lungs were divided into 3 regions (cranioventral, caudodorsal, caudoventral) and each region was assessed for the presence of 4 radiographic patterns (vascular, alveolar, interstitial, bronchial). A score of 0 was assigned when regions were considered “not different/normal” or a pattern was absent, a score of 1 for “moderate presence” of a pattern, and a score of 2 was assigned for “marked presence” of a pattern, allowing for a total score range of 0-24.

### Scoring of ultrasonographic findings

**Figure 1:**
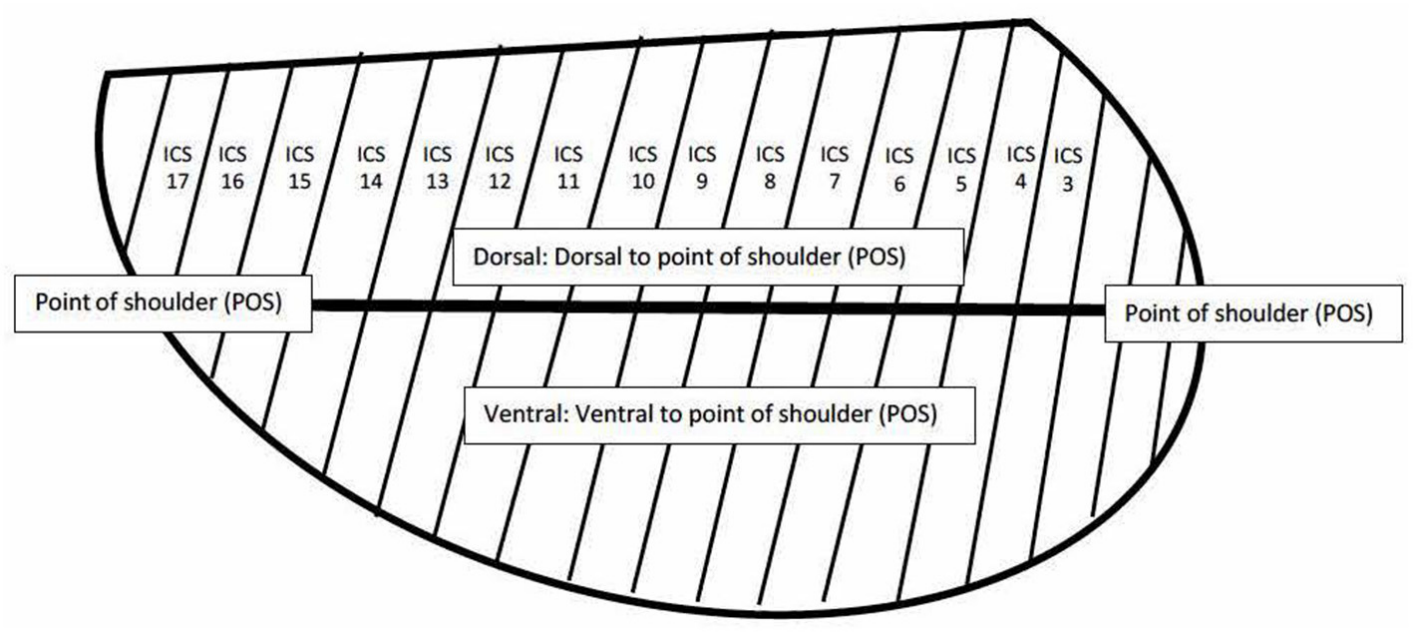
Diagram depicting intercostal spaces and dorsal and ventral regions for LUS score.

The initial ultrasonographic examination on each horse was performed by a board-certified internal medicine specialist (KHW) and a subset of horses were evaluated by a second board-certified internal medicine specialist (KHW) blinded to the results of the initial exam, but not to the group assignment of the horse. Examinations performed by two investigators were performed within 6 h of one another. Ultrasonography was performed by scanning each intercostal space (ICS) from dorsal to ventral with a 3.5 MHz curvilinear probe in B-mode with depth adjusted on a case-by-case basis. Each examination was performed with one of two identical machines (Sonoscape S9, Universal Imaging, Bedford Hills, New York, United States). Each ICS was assessed for the presence of comet tail artifacts >5 mm in length from the pleural surface, consolidation, and pleural fluid in the dorsal and ventral portions of the thorax, which were divided by a horizontal line extending caudally from the level of the point of the shoulder (see Figure 1). Comet tails were defined as linear hyperechoic artifacts perpendicular to the pleural surface. Comet tail lesions identified deep to a consolidated region of lung were also quantified. Consolidation was defined as any section of lung where the normal hyperechoic pleura was interrupted, and the tissue was hypoechoic or heterogenous in appearance. An ICS was documented as having pleural fluid if there was expansion with anechoic or hypoechoic fluid noted between the pleura and body wall. If comet tail artifacts were present, they were counted in each ICS and the number recorded. Consolidation and pleural fluid were weighted more heavily than comet tails due to their association with more severe disease and each was given a score of 4 if present and 0 if absent in an ICS (1, 22). In order to maintain ease of use for the TUS score, the weighted score did not take into account the depth of pleural effusion. However, it is the clinical impression of the authors that significant effusion would be more likely to be present in multiple ICS and in both dorsal and ventral subsections and as such would be reflected in the overall score. If lung was not visible in a portion of an ICS, no number was assigned. The score for each hemithorax was totaled for each side, and as an overall score for each horse.

### Statistical analysis

Statistical analysis was performed with R software (version 4.1.2, R foundation for Statistical Computing, Vienna, Austria). Age was examined *via* Mann-Whitney-Wilcoxon test while the gender was evaluated *via* Fisher's exact test. Differences in TUS and radiographic scores between control and diseased patients were examined *via* Mann-Whitney-Wilcoxon test. Spearman correlations were used for correlations between scores from different regions of the thorax and total ultrasonographic scores and for correlations between age and total ultrasonographic score where appropriate based on examination of plots for linear trend and homoscedasticity. Reported correlations were Pearson correlations calculated after the removal of extreme outliers. Comparisons of Pearson correlations were done using the Z transformation. The optimal cut-off for the receiver operating characteristics (ROC) analysis of ultrasound scores was determined by the concordance probability method with sensitivity and specificity reported at that point (23). Comparisons of sensitivity and specificity between ultrasound and radiography were made *via* the exact McNemar's test. Intra-class correlation coefficients used a two-way model with random effects for both subjects and raters.

## Results

### Patient demographics

The control group included 13 horses, 3 mares and 10 geldings, with a median age of 10 years (range: 6–19 years). In the diseased group there were 11 separate exams performed on 9 horses. Two horses in the diseased group were included twice as they were examined multiple weeks apart as part of disease monitoring but were treated as separate horses for purpose of analysis of the imaging findings, but only included once in analysis of signalment data. The diseased group included 1 stallion, 4 mares, and 4 geldings with a median age of 9 years (range: 8–21 years) There were no significant differences in age (*p* = 0.81) or gender (*p* = 0.19) between the two groups.

### Ultrasonographic and radiographic scores

**Figure 2:**
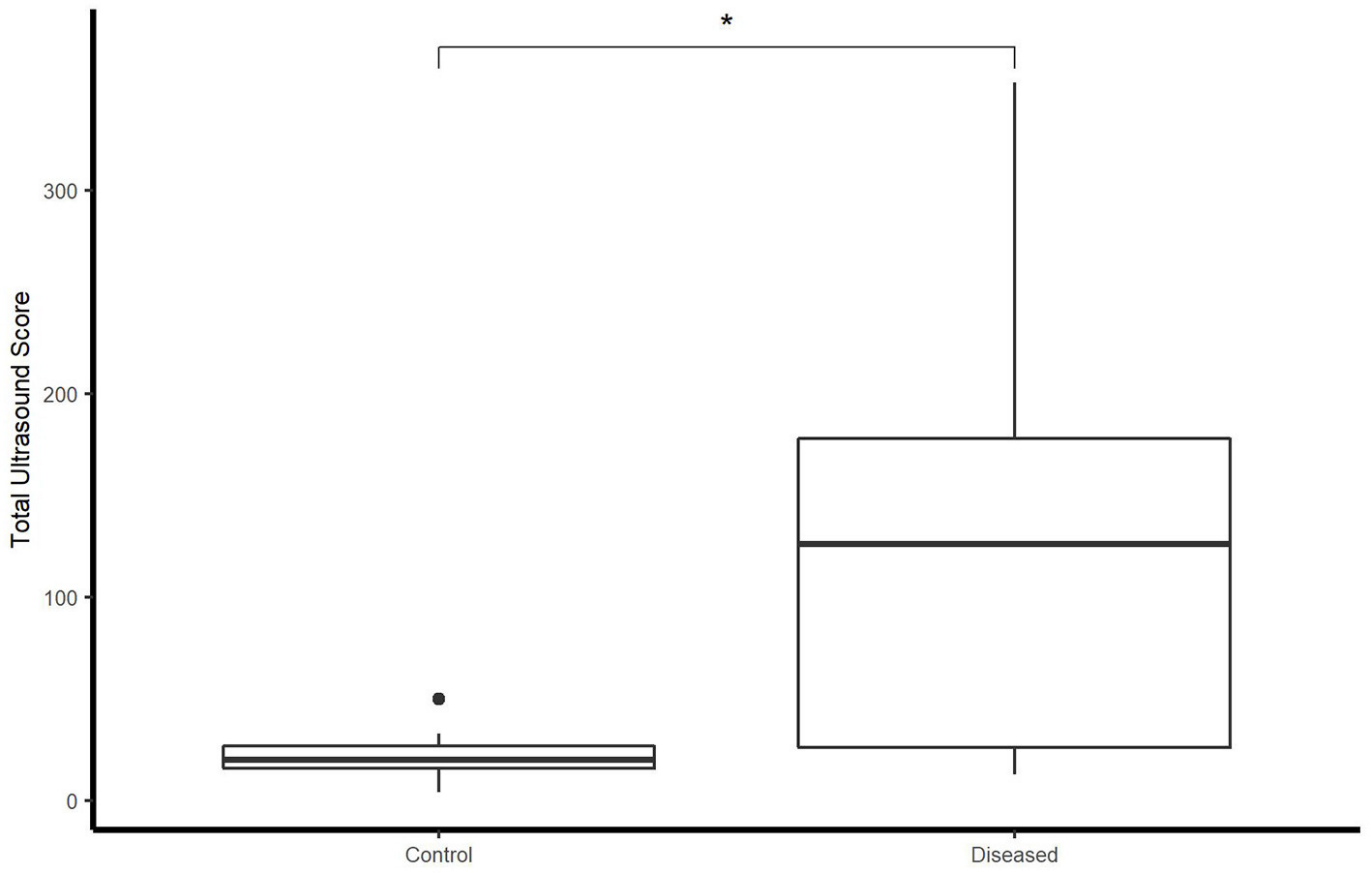
Boxplot illustrating LUS score between control and diseased group. *indicates statistical significance. Significance *p* < 0.05.

**Figure 3:**
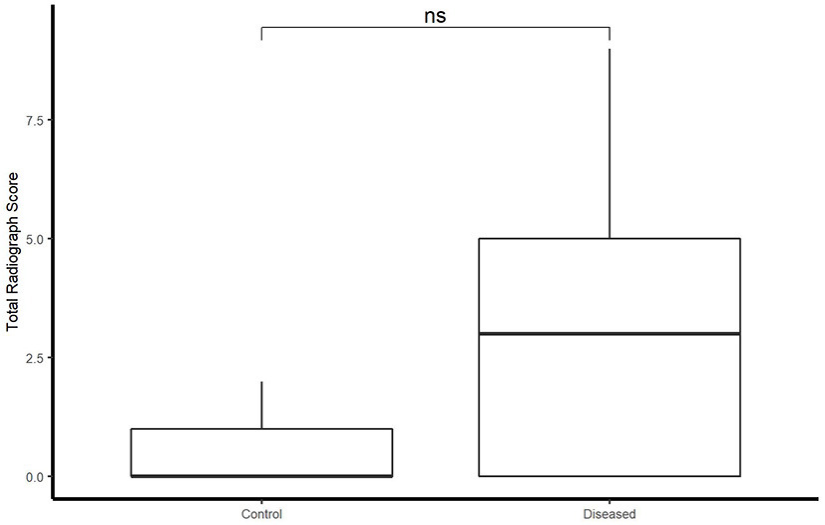
Boxplot illustrating radiograph score between control and diseased group. Significance *p* < 0.05.

**Figure 4:**
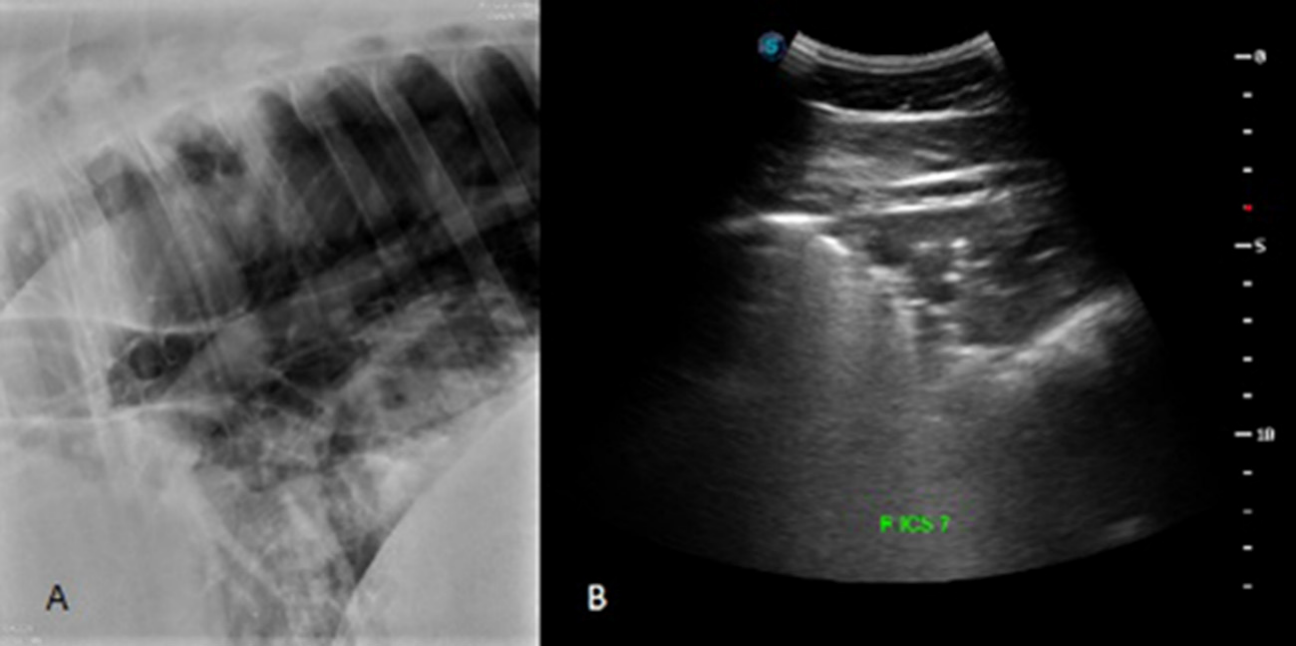
Radiographic **(A)** and ultrasonographic **(B)** image from corresponding sites on a patient in the diseased group. In the caudoventral portion of the lung, the radiograph was classified as having normal/not different presence of a vascular pattern (score of 0), a moderate presence for interstitial and alveolar patterns (score of 1 for each pattern), and a marked presence of a bronchial pattern (score of 2). The total radiographic score for this patient was 9. The ventral portion of right ICS 7 was given a comet tail score of 3 and a score of 4 for the presence of pulmonary consolidation. The total US score for this horse was 283.

**Figure 5:**
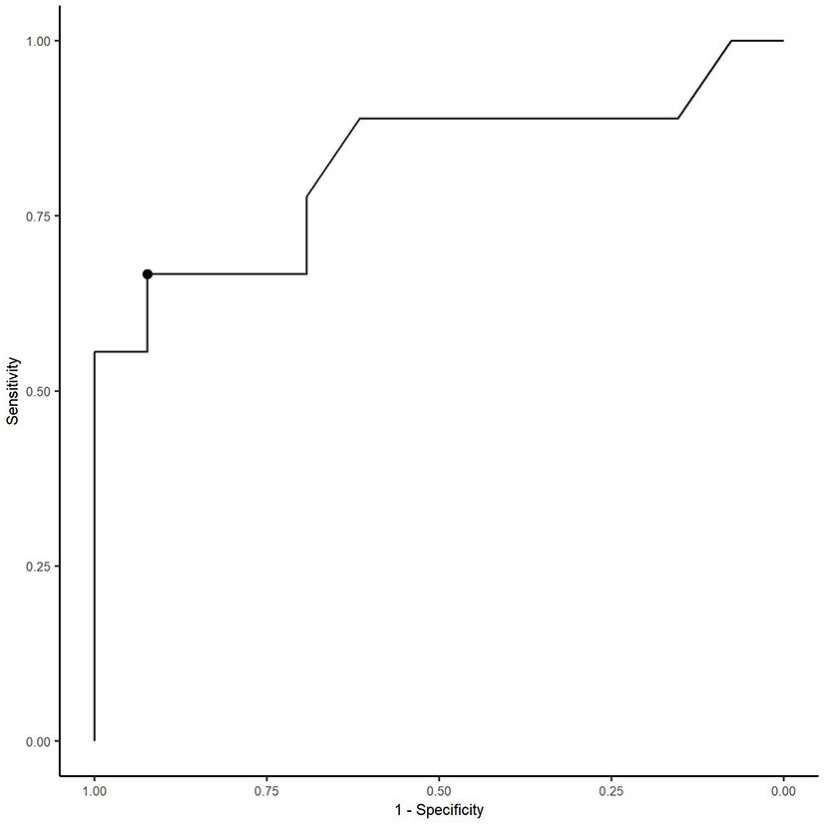
Receiver operating characteristic curve for LUS score to predict presence or absence of lower respiratory tract disease. The optimal cutoff score of 37 gave a sensitivity of 67% and specificity of 92.3%.

Total ultrasonographic score was significantly higher (*p* = 0.01) in the diseased group (median score 126; range: 13–353) than the control group (median score 20; range: 4–50) (see Figure 2). All horses in the control group had thoracic radiographs, and 5 horses in the diseased group. There was no significant difference in total radiographic score between the diseased group (median score 3; range: 0–9) and the control group (median score 0, range: 0–2) (*p* = 0.12) (see Figure 3). Ultrasonographic and radiographic scores for each individual horse are reported in Table 1. There was not a significant correlation between ultrasonographic and radiographic scores in the entire group (Pearson correlation: 0.4, *p* = 0.11, 95% CI −0.09–0.74), nor in the diseased group (Pearson correlation: 0.4, *p* = 0.60, 95% CI −0.91–0.98) or the control group (Pearson correlation: 0.53, *p* = 0.07, 95% CI −0.03–0.84). Interobserver agreement in assignment of ultrasonographic scores was good at 0.79. ROC analysis identified a sensitivity of 67% (95% CI 0.333–1) and specificity of 92.3% (95% CI 0.462–1) for the identification of bacterial pneumonia utilizing an ultrasonographic score cutoff of 37 (see Figure 4). This score was applied retroactively to allow for the calculation of accuracy. Accuracy of the ultrasonographic score when compared to clinical diagnosis was 81.8% (95% CI 0.597–0.948). One patient in the control group scored >37 (patient 2), and 3/11 exams in the diseased group scored below 37 (patients 14, 15, 17). Using an ROC cutoff of 2.5 for radiographic score sensitivity was maximized at 57.1% (95% CI 0.286–0.857) and specificity was 100% (95% CI 0.23–1). There was not a significant difference between sensitivity (*p* = 1) and specificity of the ultrasonographic or radiographic scores (*p* = 1) when applying the cutoff values from ROC analysis. See Figure 5 for example of ultrasonographic and radiographic image of a patient in the study.

**Table 1:** Ultrasonographic and radiographic findings for controls (healthy horses) and diseased horses (horses with bacterial pneumonia).

	**Control**	**Diseased**
**Ultrasonographic scores median (range)**	
Total US score	20 (4–50)	126 (13–353)
Comet tail score	20 (4–48)	65 (13–126)
Consolidation score	0 (0–2)	16 (0–140)
Pleural effusion score	0 (0–0)	0 (0–148)
**Frequency of specific ultrasonographic lesions** ***n*** **(% of total)**	
# of ultrasonographic exams with comet tails	13/13 (100%)	13/13 (100%)
# of exams with consolidation	1/13 (7.7%)	8/11 (72.7%)
# of exams with pleural effusion	0/13 (0%)	3/11 (27.3%)
**Radiographic scores median (range)**	
Total radiographic score	0 (0–2)	3 (0–9)
**Frequency of specific radiographic lesions** ***n*** **(% of total)**	
# of exams with presence of vascular pattern	0/13 (0%)	0/5 (0%)
# of exams with presence of interstitial pattern	3/13 (23.1%)	4/5 (80.0%)
# of exams with presence of alveolar pattern	0/13 (0%)	2/5 (40.0%)
# of exams with presence of bronchial pattern	2/13 (15.4%)	4/5 (80.0%)

The cranial and ventral portions of each hemi-thorax tended to be more strongly correlated with the overall score for each side and the total TUS than more caudal and dorsal sub-sections (see Table 2 for all correlations).

**Table 2:** Correlation coefficients between specific subsections of each hemithorax and the total overall thoracic ultrasonographic score.

**Sub-section**	**LV ICS** ** 3–9**	**LV ICS 10–17**	**LD ICS 3–9**	**LD ICS 10–17**	**LV ICS** ** 3–8**	**LV ICS** ** 9–12**	**LV ICS 13–17**	**LD ICS 3–8**	**LD ICS 9–12**	**LD ICS 13–17**
Correlation coefficient with left-sided score	0.85	0.36	0.74	0.51	0.87	0.48	___	0.72	0.52	0.14
Correlation coefficient with total score	0.75	0.38	0.72	0.64	0.75	0.51	___	0.71	0.61	0.33
**Sub-section**	**RV ICS 3–9**	**RV ICS 10–17**	**RD ICS 3–9**	**RD ICS 10–17**	**RV ICS 3–8**	**RV ICS 9–12**	**RV ICS 13–17**	**RD ICS 3–8**	**RD ICS 9–12**	**RD ICS 13–17**
Correlation coefficient with right-sided score	0.88	0.36	0.79	0.25	0.88	0.69	___	0.74	0.67	0.27
Correlation coefficient with total score	0.73	0.36	0.84	0.21	0.73	0.64	___	0.80	0.67	0.18

ICS, intercostal space; LV, left ventral; LD, left dorsal; RV, right ventral; RD, right dorsal.

Comet tails were the only lesions identified in control horses, with the exception of one horse that had consolidation in 2 intercostal spaces (see Table 1 for complete individual lesion scores). Therefore, only the number of comet tails per ICS and the number of ICS that contained comet tails were compared between groups. There were significantly fewer comet tails per ICS (*p* = 0.01) in the control group (median 0.67 comet tails per ICS; range 0.16–2.04) when compared to the diseased group (median 1.62 comet tails per ICS; range 0.52–5.2). Control horses did not have a statistically significantly different number of ICS that contained comet tails when compared to the diseased group (control: 11 ICS with comet tails (range 5–17), diseased:13 ICS with comet tails (range 7–26); *p* = 0.09).

## Discussion

The ultrasonographic score developed for this study provided an objective measure for differentiating healthy horses from those with bacterial pneumonia and allowed for correlation between TUS and radiography. The ultrasonographic score had similar sensitivity and specificity for the identification of bacterial pneumonia when compared to radiographic score, suggesting that in many horses the addition of thoracic radiographs may not be necessary to identify evidence of disease.

In human medicine, TUS has been shown to be an effective standalone diagnostic modality for pulmonary disease and injury (24–27). Thoracic ultrasonography is non-invasive and convenient, does not expose patients to radiation and can easily be taught to inexperienced ultrasonographers with adequate diagnostic results (27). When thoracic ultrasonography and radiography were compared to each other and to computed tomography (CT) in 144 adult human patients with acute pneumonia ultrasonography had a significantly higher sensitivity (95%) than chest radiography (60%, *p* < 0.01) (24). Ultrasonography also had 100% accuracy in identifying patients with pneumonia whereas radiography had only 52% accuracy when each was compared to chest CT (24). Severity of lesions identified on TUS also correlate well with chest CT in patients with pneumonia caused by COVID-19 (12, 15, 28).

There have been multiple studies investigating the benefit and diagnostic accuracy of utilizing TUS as the primary imaging modality in non-equine species for the identification of cardiogenic pulmonary edema, bronchopneumonia, and trauma (2–6). Thoracic ultrasonography has high sensitivity (90%) and specificity (93%) when compared to radiography for the identification of cardiogenic pulmonary edema in dogs with chronic valvular heart disease and had higher sensitivity and specificity than thoracic radiography for the diagnosis of pulmonary contusions in dogs following motor vehicle trauma (2, 6). In Holstein calves, TUS had a sensitivity of 94% and specificity of 95% for the identification of subclinical lung lesions that were confirmed on post-mortem examination (3).

Thoracic ultrasonography and radiography have been compared in the diagnosis of *Rhodococcus equi* pneumonia in foals and pneumothorax in adult horses, but not in bacterial pneumonia in adult horses. In foals, TUS has been advocated as a screening tool for diagnosis of pulmonary abscessation caused by *R. equi* pneumonia and was an accurate alternative to radiography in two studies (7, 10). The only direct comparison to date between thoracic radiography and ultrasonography in adult horses evaluated 6 horses in which small-volume pneumothorax was experimentally induced. In that study, the diagnostic sensitivity of M-mode (84%) and 2D-mode (80%) TUS was significantly higher (*p* = 0.02 and 0.04, respectively) than thoracic radiography (48%) (8).

Several scoring systems have been developed to objectively evaluate TUS images and to quantify specific lesions in humans, small animals, dairy calves and horses (3–6, 11–18, 29, 30). The benefit of an objective scoring system is increased consistency of imaging interpretation, more rapid assessment and monitoring of clinical patients, and the ability to compare imaging modalities in research settings. Ultrasonographic scores have been employed in the COVID-19 pandemic to allow for risk assessment, triage, and monitoring of patients with pneumonia (11–15). In veterinary medicine, TUS scores have been developed for use in dogs with cardiac disease, aspiration pneumonia and thoracic trauma, and in bronchopneumonia in calves (3–6, 16). Specifically in horses, TUS scores have been developed to evaluate and describe horses with specific disease processes, including Equine Influenza Virus, *Rhodococcus equi*, and equine asthma syndrome (10, 29, 30). However, no system has been developed to specifically differentiate horses with bacterial pneumonia from healthy controls and to compare TUS to radiography and to account for multiple lesion types.

Ultrasonographic scores in other species focus heavily on the presence and number of B-lines, the artifact referred to as comet tails in equine medicine (31, 32). In the scoring system described here, the authors chose to include comet tails, pulmonary consolidation and pleural effusion to provide a more complete assessment of bacterial pneumonia. Pulmonary consolidation and pleural effusion are observed in more severe pulmonary disease, whereas comet tails may be the only observed lesions in mild disease (22). A score that involves multiple types of lesions can allow for more effective classification of disease and can identify minor changes over the course of clinical disease.

Sporadic comet tail lesions have been documented in healthy horses and were also noted in the control horses in this study (29). With the exception of one control horse that had a focal region of consolidation, the only lesions present in control horses were comet tails. In human medicine, the presence of 3 or more B-lines in one region of the lung is considered abnormal, which supports the clinical impression that a low number of comet tails can be considered normal in the horse (31, 33). Every horse in the control group of this study had at least one intercostal space where comet tail lesions were noted, but the median number of comet tails per intercostal space in the control group was 0.67 (range 0.16–2.04) and did not exceed 3. By applying the cut-off TUS score of 37, an inexperienced ultrasonographer may be able to more readily differentiate a horse with bacterial pneumonia from a healthy horse with a clinically insignificant number of comet tails.

The use of zones or standardized points of evaluation when performing TUS has been applied in human medicine and provides a more rapid and targeted method of assessment than a scan of each individual ICS in emergency settings (30, 31, 34, 35). In the data reported here, scores from the right and left dorsal and ventral ICS 3-8 regions were most highly correlated with the total score for the respective sides and the overall scores than the more caudal regions. The cranioventral region (LV ICS 3-8, RV ICS 3-8, RV ICS 3-9) score on each side was the most highly correlated with the total score for that side (correlation coefficients: R 0.88, L 0.87) suggesting that a brief, targeted examination focused on the cranioventral lung fields may provide sufficient information to rapidly diagnose horses with bacterial pneumonia in an emergency setting.

The main limitation of this study was the small number of subjects. Enrollment of subjects was dependent upon hospital caseload, so varied substantially over the enrollment period. Due to the clinical nature of this study, enrolled horses were often being treated by the investigators, and thus were aware of the diagnosis, which may have biased interpretation of ultrasonographic findings. However, by establishing an objective means of quantifying changes identified on ultrasound, this bias was reduced. Despite knowledge of the patient's diagnosis, ultrasonographers (KHW and KD) were not present for exams performed by the other ultrasonographer and were blinded to each other's scores. Two horses included in the diseased group were enrolled twice leading to a total of 11 ultrasonographic examinations in the diseased group. However, examinations were performed at least 2 weeks apart when their clinical disease was likely to have changed significantly, and earlier scores were not reviewed prior to the examination to avoid bias. Demographic data for these 2 horses was only included in analysis once. Six of 11 ultrasonographic examinations in the diseased group did not have concurrent radiographs performed, which may explain why the difference in radiographic score between healthy (median score 0) and diseased horses (median score 3) did not reach statistical significance. It is possible that with a larger number of subjects, the differences in radiographic score would be statistically significant.

The data presented here supports growing evidence in small animal and human medicine that point-of-care TUS provides a rapid and sensitive method for the diagnosis of pneumonia in adult horses without the addition of radiography. The ultrasonographic score described here was able to readily differentiate horses with confirmed bacterial pneumonia from healthy horses with a calculated accuracy of 81%. Additionally, there was no significant difference in sensitivity and specificity of ultrasonography and radiography. By utilizing the ultrasonographic scoring system described here and applying the cut-off score of 37, practitioners can readily distinguish healthy horses from those with bacterial pneumonia. Future studies are warranted to compare TUS scores between equine asthma and bacterial pneumonia to assess the utility of the score in differentiating between the two disease processes, but by quantifying multiple types of lesions (comet tails, pulmonary consolidation and pleural effusion), it is plausible that the score could be applied in the evaluation of horses presenting for evaluation of lower respiratory disease. It is important to note that TUS is unable to identify lesions that do not extend to the pleural surface. However, in bacterial pneumonia of adult horses it is rare for deep lesions, such as abscessation, to be present without accompanying comet tails, consolidation or pleural effusion (1, 22). Therefore, TUS can be recommended as an accurate method for the initial identification of bacterial pneumonia in many cases without the need for immediate additional imaging in the field setting where radiography of the thorax is often impractical.

## Data availability statement

The raw data supporting the conclusions of this article will be made available by the authors, upon reasonable request, without undue reservation.

## Ethics statement

The animal study was reviewed and approved by North Carolina State University Institutional Animal Care and Use Committee. Written informed consent was obtained from the owners for the participation of their animals in this study.

## Author contributions

KH-W, NN, and KD designed the study and ultrasonographic scoring system. KH-W performed all initial ultrasonographic examinations. KD served as second ultrasonographer. NN performed radiographic evaluations. KY assisted in data collection and examinations. All authors contributed to manuscript preparation and approved the submitted version.

## Funding

This study was funded by North Carolina State University College of Veterinary Medicine start-up funds.

## Conflict of interest

The authors declare that the research was conducted in the absence of any commercial or financial relationships that could be construed as a potential conflict of interest.

## Publisher's note

All claims expressed in this article are solely those of the authors and do not necessarily represent those of their affiliated organizations, or those of the publisher, the editors and the reviewers. Any product that may be evaluated in this article, or claim that may be made by its manufacturer, is not guaranteed or endorsed by the publisher.

## References

[1] Reuss SM Giguère S. Update on bacterial pneumonia and pleuropneumonia in the adult horse. Vet Clin North Am Equine. (2015) 31:105–20. 10.1016/j.cveq.2014.11.002 25600453

[2] Vezzosi T Mannucci T Pistoresi A Toma F Tognett R Zini E. Assessment of Lung ultrasound B-lines in dogs with different stages of chronic valvular heart disease. J Vet Intern Med. (2017) 31:700–4. 10.1111/jvim.14692 28370336 PMC5435052

[3] Ollivett TL Caswell JL Nydam DV Duffield T Leslie KE Hewson J. Thoracic ultrasonography and bronchoalveolar lavage fluid analysis in holstein calves with subclinical lung lesions. J Vet Intern Med. (2015) 29:1728–34. 10.1111/jvim.13605 26332345 PMC4895683

[4] Berman J Masseau I Fecteau G Buczinski S Francoz D. Comparison between thoracic ultrasonography and thoracic radiography for the detection of thoracic lesions in dairy calves using a two-stage bayesian method. Prev Vet Med. (2020) 184:105–53. 10.1016/j.prevetmed.2020.105153 32992242

[5] Ward JL Lisciandro GR Keene BW Tou SP DeFrancesco TC. Accuracy of point-of-care lung ultrasonography for the diagnosis of cardiogenic pulmonary edema in dogs and cats with acute dyspnea. J Am Vet Med Assoc. (2017) 250:666–75. 10.2460/javma.250.6.666 28263112

[6] Dicker SA Lisciandro GR Newell SM Johnson JA. Diagnosis of pulmonary contusions with point-of-care lung ultrasonography and thoracic radiography compared to thoracic computed tomography in dogs with motor vehicle trauma: 29 cases (2017-2018). J Vet Emerg Crit Care. (2020) 30:638–46. 10.1111/vec.13021 33085212

[7] Ramirez S Lester GD Roberts GR. Diagnostic contribution of thoracic ultrasonography in 17 foals with rhodococcus equi pneumonia. Vet Radiol Ultrasound. (2004) 45:172–6. 10.1111/j.1740-8261.2004.04028.x 15072151

[8] Partlow J David F Hunt LM Relave F Blond L Pinilaa M. Comparison of thoracic ultrasonography and radiography for the detection of induced small volume pneumothorax in the horse. Vet Radiol Ultrasound. (2017) 58:354–60. 10.1111/vru.12480 28264227

[9] Giguère S Roberts GD. Association between radiographic pattern and outcome in foals with pneumonia caused by rhodococcus equi. Vet Radiol Ultrasound. (2012) 53:601–4. 10.1111/j.1740-8261.2012.01964.x 22742474

[10] McCracken JL Slovis NM. Use of Thoracic Ultrasound for the Prevention of Rhodococcus equi Pneumonia on Endemic Farms. In: Proceedings of the 55th Annual Convention of the American Association of Equine Practitioners. (2009). Las Vegas, NV.

[11] Dargent A Chatelain E Kreitmann L Quenot JP Cour M Argaud L. Lung Ultrasound score to monitor COVID-19 pneumonia progression in patients with ARDS. PLoS ONE. (2020) 15:1–4. 10.1371/journal.pone.0236312 32692769 PMC7373285

[12] Zieleskiewicz L Markarian T Lopez A Taguet C Mohammedi N Boucekine M. Comparative Study of Lung Ultrasound and Chest Computed Tomography Scan in the Assessment of Severity of Confirmed COVID-19 Pneumonia. Intensive Care Med. (2020) 46:1707–13. 10.1007/s00134-020-06186-0 32728966 PMC7388119

[13] Ji L Cao C Gao Y Zhang W Xie Y Duan Y. Prognostic value of bedside lung ultrasound score in patients with COVID-19. Crit Care. (2020) 24:1–12. 10.1186/s13054-020-03416-1 33353548 PMC7754180

[14] Alencar de Julio Cesar G Marchini JFM Marino LO da Costa Ribeiro SC Bueno CG. Lung ultrasound score predicts outcomes in COVID-19 patients admitted to the emergency department. Ann Intensive Care. (2021) 11:6. 10.1186/s13613-020-00799-w 33427998 PMC7797883

[15] Nouvenne A Zani M Milanese G Parrise A Baciarello M Bignami EG. Lung ultrasound in COVID-19 pneumonia: correlations with chest CT on hospital admission. Respir. (2020) 99:617–24. 10.1159/000509223 32570265 PMC7360505

[16] Rodrigues NF Giraud L Bolen G Fastres A Clercx C Boysen S. Comparison of lung ultrasound, chest radiographs, c-reactive protein, and clinical findings in dogs treated for aspiration pneumonia. J Vet Intern Med. (2022) 36:1–10. 10.1111/jvim.16379 35247005 PMC8965265

[17] Ferrucci F Stancari G Zucca E Ayalon S Falcone C Ferro E. Specificity and sensitivity of ultrasonography and endoscopy for the diagnosis of exercise-induced pulmonary haemorrhage (EIPH) in 157 race horses. Vet Res Commun. (2009) 33:5185–8. 10.1007/s11259-009-9277-5 19578953

[18] Lo Feudo CM Stucchi L Alberti E Stancari G Conurba B Zucca E. The role of thoracic ultrasonography and airway endoscopy in the diagnosis of equine asthma and exercise-induced pulmonary hemorrhage. Vet Sci. (2021) 8:1–15. 10.3390/vetsci8110276 34822649 PMC8619806

[19] Lichter Y Topilsky Y Taieb P Banai A Hochstadt A Merdler I. Lung Ultrasound predicts clinical course and outcomes in COVID-19 patients. Intensive Care Med. (2020) 46:1873–83. 10.1007/s00134-020-06212-1 32860069 PMC7454549

[20] Brattain LJ Telfer BA Liteplo AS Noble VE. Automated B-line scoring on thoracic sonography. J Med Ultrasound. (2013) 32:2185–90. 10.7863/ultra.32.12.2185 24277902

[21] Mazan MR Vin R Hoffman AM. Radiographic scoring lacks predictive value in inflammatory airway disease. Equine Vet J. (2005) 37:541–5. 10.2746/042516405775314899 16295932

[22] Ferrucci F Zucca E Croci C Di Fabio V Martino PA Ferro E. Bacterial pneumonia and pleuropneumonia in sport horses: 17 Cases (2001–2003). Equine Vet Educ. (2008) 20:526–31. 10.2746/095777308X354255

[23] Liu X. Classification accuracy and cut point selection. Stat Med. (2012) 31:2676–86. 10.1002/sim.4509 22307964

[24] Bourcier J Paquet J Seinger M Gallard E Redonnet JP Cheddadi F. Performance comparison of lung ultrasound and chest X-ray for the diagnosis of pneumonia in the ED. Am J Emerg Med. (2014) 32:115–8. 10.1016/j.ajem.2013.10.003 24184011

[25] Long L Zhao HT Zhang ZY Wang GY Zhao HL. Lung ultrasound for the diagnosis of pneumonia in adults. Medicine. (2017) 96:e5713. 10.1097/MD.0000000000005713 28099332 PMC5279077

[26] Hu QJ Shen YC Jia LQ Guo SJ Long HY Pang CS. Diagnostic performance of lung ultrasound in the diagnosis of pneumonia: a bivariate meta-analysis. Int J Clin Exp Med. (2014) 7:115–21. 24482696 PMC3902248

[27] Jones BP Tay ET Elikashvili I Sanders JE Paul AZ Nelson BP. Feasibility and safety of substituting lung ultrasonography for chest radiography when diagnosing pneumonia in children. Chest. (2016) 150:131–8. 10.1016/j.chest.2016.02.643 26923626

[28] Deng Q Cao S Wang H Zhang Y Chen L Yang Z. Application of quantitative lung ultrasound instead of CT for monitoring COVID-19 pneumonia in pregnant women: a single-center retrospective study. BMC Pregnancy Childbirth. (2021) 21:259. 10.1186/s12884-021-03728-2 33771120 PMC7997654

[29] Siwinska N Zak A Slowikowska M Krupinska P Niedzwiedz A. Prevalence and severity of ultrasonographic pulmonary findings in horses with asthma - a preliminary study. Pol J Vet Sci. (2019) 22:653–9. 10.24425/pjvs.2019.129977 31867937

[30] Gross DK Morley PS Hinchcliff KW Reichle JK Slemons RD. Pulmonary Ultrasonographic abnormalities associated with naturally occurring equine influenza virus infection in standardbred racehorses. J Vet Intern Med. (2004) 18:718–27. 10.1111/j.1939-1676.2004.tb02611.x 15515590

[31] Gullett J Donnelly JP Sinert R Hosek B Fuller D Hill H. Interobserver agreement in the evaluation of B-lines using bedside ultrasound. J Crit Care. (2015) 30:1395–9. 10.1016/j.jcrc.2015.08.021 26404955

[32] Wang Y Zhang Y He Q Liao H Luo J. Quantitative analysis of pleural line and B-lines in lung ultrasound images for severity assessment of COVID-19 pneumonia. IEEE Trans Ultrason Ferroelectr Freq Control. (2022) 69:73–83. 10.1109/TUFFC.2021.3107598 34428140 PMC8905613

[33] Rambhia SH D'Agostino CA Noor A Villani R Naidich JJ Pellerito JS. Thoracic ultrasound: technique, applications, and interpretation. Curr Probl Diagn Radiol. (2016) 46:305–16. 10.1067/j.cpradiol.2016.12.003 28185691

[34] Volpicelli G Elbarbary M Blaivas M Lichtenstein DA Mathis G Kirkpatrick AW. International evidence-based recommendations for point-of-care lung ultrasound. Intensive Care Med. (2012) 38:577–91. 10.1007/s00134-012-2513-4 22392031

[35] Lichtenstein D. BLUE-protocol and FALLS-protocol. Chest. (2015) 147:1659–70. 10.1378/chest.14-1313 26033127

